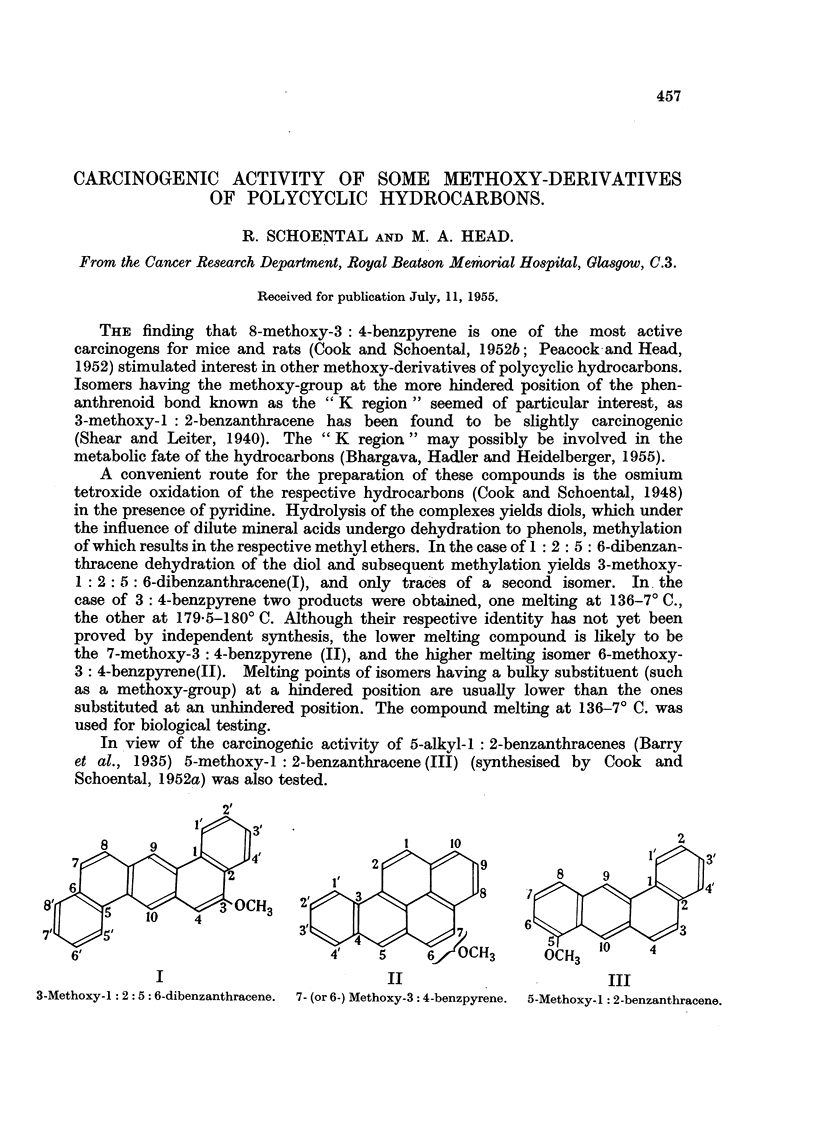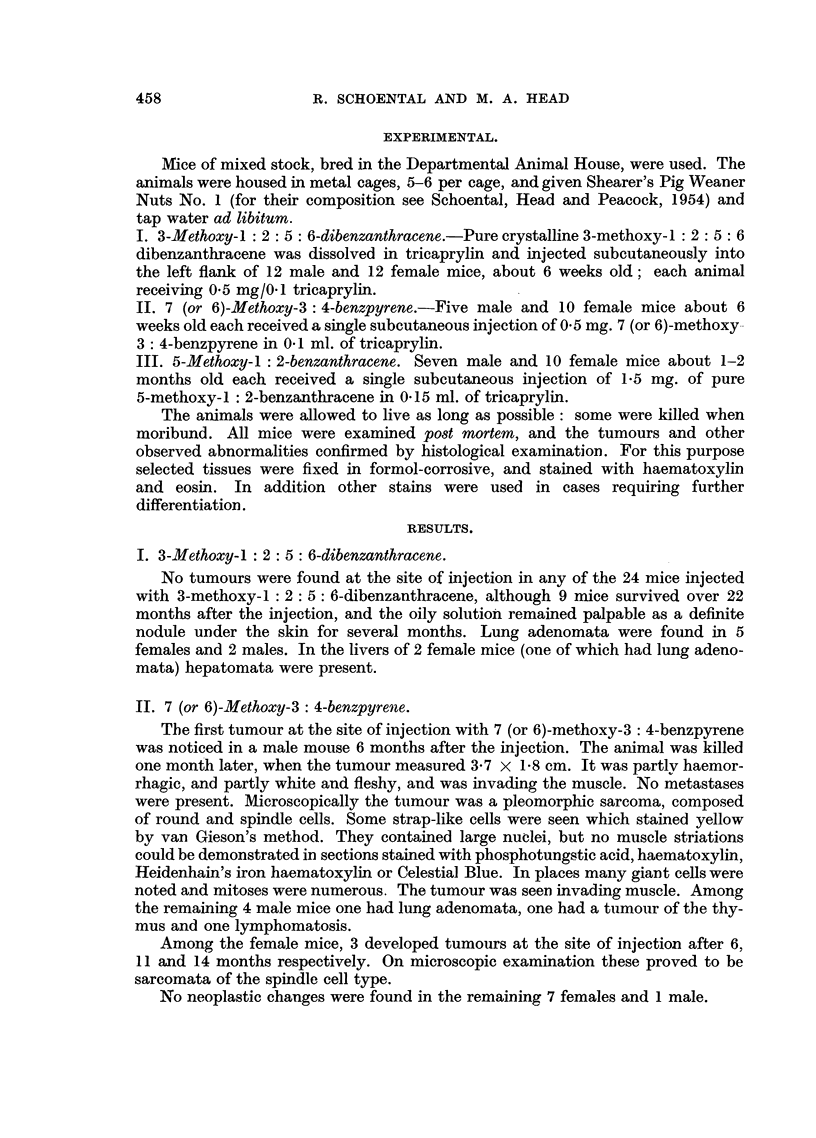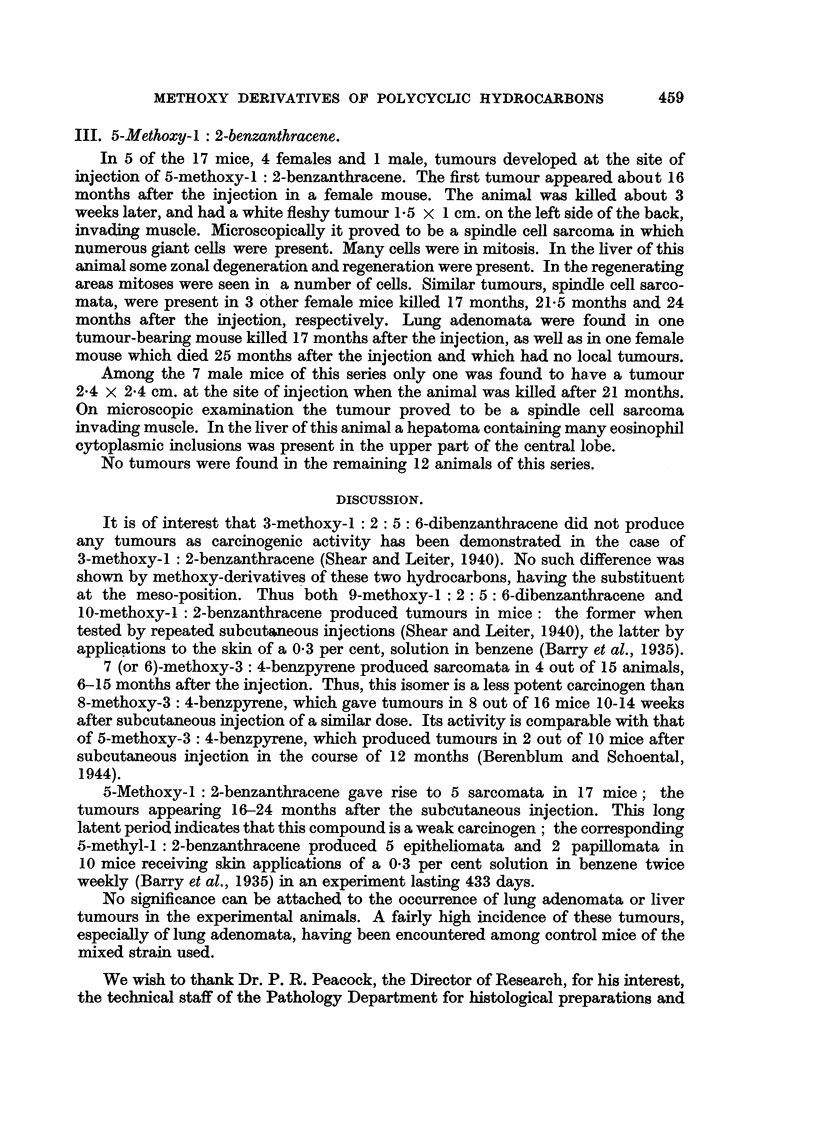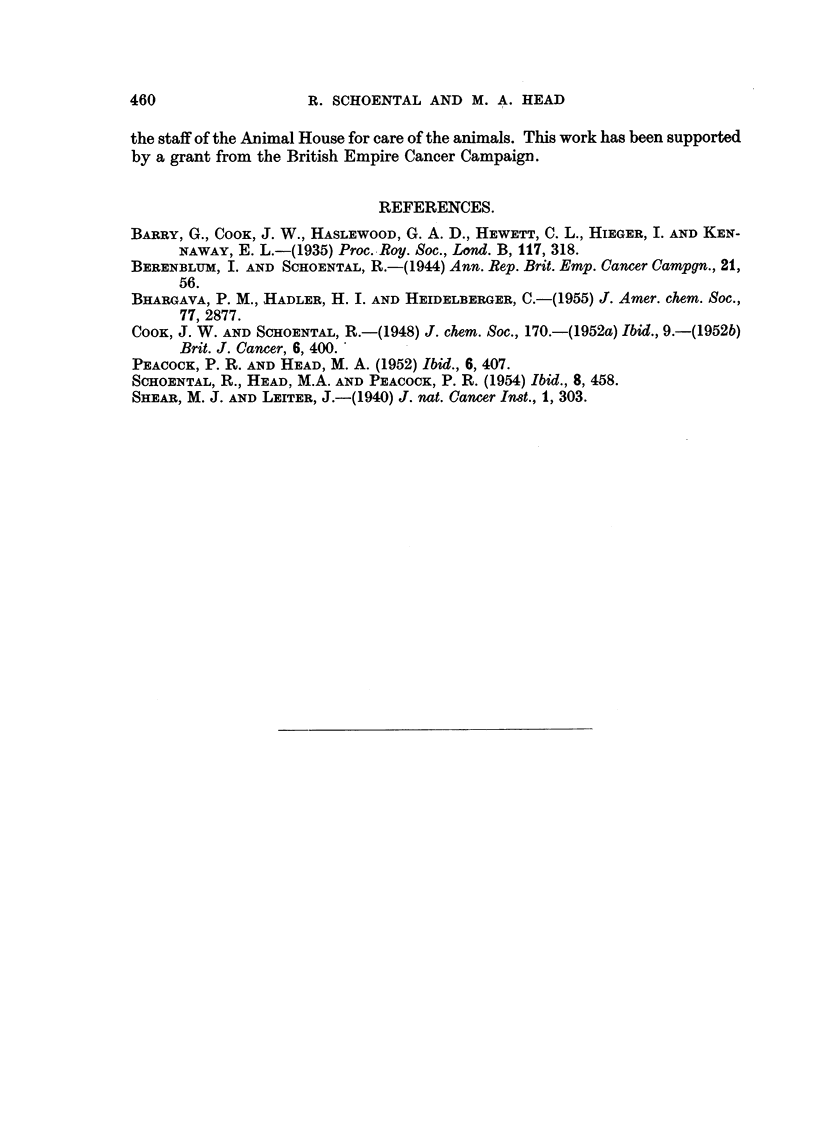# Carcinogenic Activity of Some Methoxyderivatives of Polycyclic Hydrocarbons

**DOI:** 10.1038/bjc.1955.46

**Published:** 1955-09

**Authors:** R. Schoental, M. A. Head


### CARCINOGENIC ACTIVITY OF SOME METHOXY-DERIVATIVES OF POLYCYCLIC HYDROCARBONS.

R. SCHOENTAL AND M. A. HEAD.

*From the Cancer Research Department, Royal Beatson Memorial Hospital, Glasgow, C.3.*

Received for publication July, 11, 1955.

THE finding that 8-methoxy-3 : 4-benzpyrene is one of the most active carcinogens for mice and rats (Cook and Schoental, 1952*b*; Peacock and Head, 1952) stimulated interest in other methoxy-derivatives of polycyclic hydrocarbons. Isomers having the methoxy-group at the more hindered position of the phenanthrenoid bond known as the " K region " seemed of particular interest, as 3-methoxy-1 : 2-benzanthracene has been found to be slightly carcinogenic (Shear and Leiter, 1940). The " K region " may possibly be involved in the metabolic fate of the hydrocarbons (Bhargava, Hadler and Heidelberger, 1955).

A convenient route for the preparation of these compounds is the osmium tetroxide oxidation of the respective hydrocarbons (Cook and Schoental, 1948) in the presence of pyridine. Hydrolysis of the complexes yields diols, which under the influence of dilute mineral acids undergo dehydration to phenols, methylation of which results in the respective methyl ethers. In the case of 1 : 2 : 5 : 6-dibenzanthracene dehydration of the diol and subsequent methylation yields 3-methoxy-1 : 2 : 5 : 6-dibenzanthracene(I), and only traces of a second isomer. In the case of 3 : 4-benzpyrene two products were obtained, one melting at 136–7° C., the other at 179·5–180° C. Although their respective identity has not yet been proved by independent synthesis, the lower melting compound is likely to be the 7-methoxy-3 : 4-benzpyrene (II), and the higher melting isomer 6-methoxy-3 : 4-benzpyrene(II). Melting points of isomers having a bulky substituent (such as a methoxy-group) at a hindered position are usually lower than the ones substituted at an unhindered position. The compound melting at 136–7° C. was used for biological testing.

In view of the carcinogenic activity of 5-alkyl-1 : 2-benzanthracenes (Barry *et al.*, 1935) 5-methoxy-1 : 2-benzanthracene (III) (synthesised by Cook and Schoental, 1952*a*) was also tested.

I

3-Methoxy-1 : 2 : 5 : 6-dibenzanthracene.

II

7- (or 6-) Methoxy-3 : 4-benzpyrene.

III

5-Methoxy-1 : 2-benzanthracene.

EXPERIMENTAL.

Mice of mixed stock, bred in the Departmental Animal House, were used. The animals were housed in metal cages, 5–6 per cage, and given Shearer's Pig Weaner Nuts No. 1 (for their composition see Schoental, Head and Peacock, 1954) and tap water *ad libitum.*

I. 3-*Methoxy*-1 : 2 : 5 : 6-*dibenzanthracene.*—Pure crystalline 3-methoxy-1 : 2 : 5 : 6 dibenzanthracene was dissolved in tricaprylin and injected subcutaneously into the left flank of 12 male and 12 female mice, about 6 weeks old ; each animal receiving 0·5 mg/0·1 tricaprylin.

II. 7 (*or* 6)-*Methoxy*-3 : 4-*benzpyrene.*—Five male and 10 female mice about 6 weeks old each received a single subcutaneous injection of 0·5 mg. 7 (or 6)-methoxy-3 : 4-benzpyrene in 0·1 ml. of tricaprylin.

III. 5-*Methoxy*-1 : 2-*benzanthracene.* Seven male and 10 female mice about 1–2 months old each received a single subcutaneous injection of 1·5 mg. of pure 5-methoxy-1 : 2-benzanthracene in 0·15 ml. of tricaprylin.

The animals were allowed to live as long as possible : some were killed when moribund. All mice were examined *post mortem,* and the tumours and other observed abnormalities confirmed by histological examination. For this purpose selected tissues were fixed in formol-corrosive, and stained with haematoxylin and eosin. In addition other stains were used in cases requiring further differentiation.

RESULTS.

I. 3-*Methoxy*-1 : 2 : 5 : 6-*dibenzanthracene.*

No tumours were found at the site of injection in any of the 24 mice injected with 3-methoxy-1 : 2 : 5 : 6-dibenzanthracene, although 9 mice survived over 22 months after the injection, and the oily solution remained palpable as a definite nodule under the skin for several months. Lung adenomata were found in 5 females and 2 males. In the livers of 2 female mice (one of which had lung adenomata) hepatomata were present.

II. 7 (*or* 6)-*Methoxy*-3 : 4-*benzpyrene.*

The first tumour at the site of injection with 7 (or 6)-methoxy-3 : 4-benzpyrene was noticed in a male mouse 6 months after the injection. The animal was killed one month later, when the tumour measured 3·7 × 1·8 cm. It was partly haemorrhagic, and partly white and fleshy, and was invading the muscle. No metastases were present. Microscopically the tumour was a pleomorphic sarcoma, composed of round and spindle cells. Some strap-like cells were seen which stained yellow by van Gieson's method. They contained large nuclei, but no muscle striations could be demonstrated in sections stained with phosphotungstic acid, haematoxylin, Heidenhain's iron haematoxylin or Celestial Blue. In places many giant cells were noted and mitoses were numerous. The tumour was seen invading muscle. Among the remaining 4 male mice one had lung adenomata, one had a tumour of the thymus and one lymphomatosis.

Among the female mice, 3 developed tumours at the site of injection after 6, 11 and 14 months respectively. On microscopic examination these proved to be sarcomata of the spindle cell type.

No neoplastic changes were found in the remaining 7 females and 1 male.

III. 5-*Methoxy*-1 : 2-*benzanthracene*.

In 5 of the 17 mice, 4 females and 1 male, tumours developed at the site of injection of 5-methoxy-1 : 2-benzanthracene. The first tumour appeared about 16 months after the injection in a female mouse. The animal was killed about 3 weeks later, and had a white fleshy tumour 1·5 × 1 cm. on the left side of the back, invading muscle. Microscopically it proved to be a spindle cell sarcoma in which numerous giant cells were present. Many cells were in mitosis. In the liver of this animal some zonal degeneration and regeneration were present. In the regenerating areas mitoses were seen in a number of cells. Similar tumours, spindle cell sarcomata, were present in 3 other female mice killed 17 months, 21·5 months and 24 months after the injection, respectively. Lung adenomata were found in one tumour-bearing mouse killed 17 months after the injection, as well as in one female mouse which died 25 months after the injection and which had no local tumours.

Among the 7 male mice of this series only one was found to have a tumour 2·4 × 2·4 cm. at the site of injection when the animal was killed after 21 months. On microscopic examination the tumour proved to be a spindle cell sarcoma invading muscle. In the liver of this animal a hepatoma containing many eosinophil cytoplasmic inclusions was present in the upper part of the central lobe.

No tumours were found in the remaining 12 animals of this series.

### DISCUSSION.

It is of interest that 3-methoxy-1 : 2 : 5 : 6-dibenzanthracene did not produce any tumours as carcinogenic activity has been demonstrated in the case of 3-methoxy-1 : 2-benzanthracene (Shear and Leiter, 1940). No such difference was shown by methoxy-derivatives of these two hydrocarbons, having the substituent at the meso-position. Thus both 9-methoxy-1 : 2 : 5 : 6-dibenzanthracene and 10-methoxy-1 : 2-benzanthracene produced tumours in mice : the former when tested by repeated subcutaneous injections (Shear and Leiter, 1940), the latter by applications to the skin of a 0·3 per cent, solution in benzene (Barry *et al.*, 1935).

7 (or 6)-methoxy-3 : 4-benzpyrene produced sarcomata in 4 out of 15 animals, 6–15 months after the injection. Thus, this isomer is a less potent carcinogen than 8-methoxy-3 : 4-benzpyrene, which gave tumours in 8 out of 16 mice 10-14 weeks after subcutaneous injection of a similar dose. Its activity is comparable with that of 5-methoxy-3 : 4-benzpyrene, which produced tumours in 2 out of 10 mice after subcutaneous injection in the course of 12 months (Berenblum and Schoental, 1944).

5-Methoxy-1 : 2-benzanthracene gave rise to 5 sarcomata in 17 mice ; the tumours appearing 16–24 months after the subcutaneous injection. This long latent period indicates that this compound is a weak carcinogen ; the corresponding 5-methyl-1 : 2-benzanthracene produced 5 epitheliomata and 2 papillomata in 10 mice receiving skin applications of a 0·3 per cent solution in benzene twice weekly (Barry *et al.*, 1935) in an experiment lasting 433 days.

No significance can be attached to the occurrence of lung adenomata or liver tumours in the experimental animals. A fairly high incidence of these tumours, especially of lung adenomata, having been encountered among control mice of the mixed strain used.

We wish to thank Dr. P. R. Peacock, the Director of Research, for his interest, the technical staff of the Pathology Department for histological preparations and the staff of the Animal House for care of the animals. This work has been supported by a grant from the British Empire Cancer Campaign.

### REFERENCES.

BARRY, G., COOK, J. W., HASLEWOOD, G. A. D., HEWETT, C. L., HIEGER, I. AND KENNAWAY, E. L.—(1935) *Proc. Roy. Soc., Lond.* B, **117**, 318.

BERENBLUM, I. AND SCHOENTAL, R.—(1944) *Ann. Rep. Brit. Emp. Cancer Campgn.*, **21**, 56.

BHARGAVA, P. M., HADLER, H. I. AND HEIDELBERGER, C.—(1955) *J. Amer. chem. Soc.*, **77**, 2877.

COOK, J. W. AND SCHOENTAL, R.—(1948) *J. chem. Soc.*, 170.—(1952*a*) *Ibid.*, 9.—(1952*b*) *Brit. J. Cancer*, **6**, 400.

PEACOCK, P. R. AND HEAD, M. A. (1952) *Ibid.*, **6**, 407.

SCHOENTAL, R., HEAD, M.A. AND PEACOCK, P. R. (1954) *Ibid.*, **8**, 458.

SHEAR, M. J. AND LEITER, J.—(1940) *J. nat. Cancer Inst.*, **1**, 303.